# Comparative Analysis of Lower Genital Tract Microbiome Between PCOS and Healthy Women

**DOI:** 10.3389/fphys.2020.01108

**Published:** 2020-09-08

**Authors:** Yaoyao Tu, Guangyong Zheng, Guolian Ding, Yanting Wu, Ji Xi, Yingzhou Ge, Hangchao Gu, Yingyu Wang, Jianzhong Sheng, Xinmei Liu, Li Jin, Hefeng Huang

**Affiliations:** ^1^Department of Obstetrics and Gynecology, International Peace Maternity and Child Health Hospital, Shanghai Jiao Tong University, Shanghai, China; ^2^Shanghai Key Laboratory of Embryo Original Diseases, Shanghai, China; ^3^Bio-Med Big Data Center, CAS Key Laboratory of Computational Biology, CAS-MPG Partner Institute for Computational Biology, Shanghai Institute of Nutrition and Health, Shanghai Institutes for Biological Sciences, Chinese Academy of Sciences, Shanghai, China; ^4^Key Laboratory of Reproductive Genetics, Ministry of Education, Zhejiang University, Hangzhou, China; ^5^Department of Pathology and Pathophysiology, School of Medicine, Zhejiang University, Hangzhou, China

**Keywords:** *Gardnerella*, *Lactobacillus*, lower genital tract (LGT), microbiome, PCOS

## Abstract

Women with polycystic ovarian syndrome (PCOS) often have a history of infertility and poor pregnancy outcome. The character of the lower genital tract (LGT) microbiome of these patients is still unknown. We collected both vaginal and cervical canal swabs from 47 PCOS patients (diagnosed by the Rotterdam Criteria) and 50 healthy reproductive-aged controls in this study. Variable regions 3–4 (V3–4) were sequenced and analyzed. Operational taxonomic unit (OTU) abundance was noted for all samples. Taxa that discriminated between PCOS and healthy women was calculated by linear discriminant analysis effect size (LEFSe). Results from 97 paired vaginal and cervical canal samples collected from 97 women [mean age 30 (±4 years)] were available for analysis. Using the Rotterdam Criteria, 47 women were diagnosed with PCOS (PCOS, *n* = 47; control, *n* = 50). There was no significant difference between cervical canal microbiome and vaginal microbiome from the same individual, however, *Lactobacillus* spp. was less abundant in both vaginal and cervical canal microbiome of PCOS patients. Several non-*Lactobacillus* taxa including *Gardnerella_vaginalis_00703mash*, *Prevotella_9_other*, and *Mycoplasma hominis*, were more abundant in the LGT microbiota of PCOS patients. There is a difference between the microorganism in the LGT of patients with PCOS and healthy reproductive-aged women.

## Introduction

Polycystic ovarian syndrome (PCOS) is one of the most common endocrine syndromes of reproductive-aged women, which displays diverse symptoms including irregular menstruation (due to oligo-ovulation or anovulation), sterility, recurrent abortion, and also metabolic disorder, affecting about 10 to 15% of women worldwide and costing millions of dollars per year. PCOS is becoming the most common cause of sterility in reproductive-aged women (Wang et al., 2015; Bellver et al., 2018; Patel, 2018).

Microbiome of female genital tract has been found to be closely related to women’s health in the last decades (Young, 2017). It is well known that the genus *Lactobacillus* is the most abundant microorganism in the vaginal, cervical, as well as endometrial areas of reproductive-aged women, the diversity of microbiota in women’s genital tract have been reported to be linked with several adverse reproductive issues such as infertility, recurrent abortion, poor outcome of IVF, and preterm birth (Sirota et al., 2014; Torcia, 2019; Tsonis et al., 2020). Recently, several researches have discovered that the gut microbiome might play a critical role in the progression of PCOS, especially in its metabolic disorder, which is highly correlated to the level of testosterone (Torres et al., 2018). However, the composition of PCOS patients’ lower genital tract microbiome has not been characterized, and the relationship between PCOS and the lower genital tract (LGT) microbiome has also not been studied yet. Discovering the situation of PCOS women’s LGT microbiome may help us obtain a more comprehensive understanding of this syndrome, and by the angle of lower genital tract microenvironment, may provide new insights into the mechanism and treatment on the reproductive issues of PCOS patients.

Here, we sampled microbiota biopsy of both the vagina and cervical canal of 97 reproductive-aged women through a less invasive method. The total of 194 microbial samples were detected by 16S rRNA gene sequencing. The results indicate a significant difference of taxa abundance between PCOS and healthy women in both vaginal and cervical canal microbiome.

## Materials and Methods

### Study Population and Study Design

Ninety-seven reproductive-aged Chinese women were recruited at the International Peace Maternity and Child Health Hospital between December 2018 and June 2019. PCOS was diagnosed using the Rotterdam Criteria. Patients should present with at least two of the following: irregular menstrual periods (oligomenorrhoea or amenorrhea), polycystic ovaries, and hyperandrogenism. Cushing’s syndrome, congenital adrenal hyperplasia, thyroid disorder, hyperprolactinemia, and androgen-secreting tumors were excluded. For the controls, healthy women who were of similar age as the PCOS patients were recruited. All of them came to the assisted reproductive clinic because of male factors, and all their physical examination indexes were normal. Subjects with inflammation, endocrine disorders, or cancer were excluded. The subjects should not use hormone, antibiotic, or vaginal medicine within 7 days, cervical treatment or flushing should not be carried out within 5 days, and sexual behavior should not be carried out within 48 h. Women who were pregnant, nursing, or menstruating at the time of sampling were also excluded (Chen et al., 2017). All patients should provide a written informed consent. This study was approved by the International Peace Maternity and Child Health Hospital (ethics approval number GKLW 2018-10).

### Sample Collection

Swabs were collected on the day of clinical visit. Vaginal swabs were collected directly, while cervical canal swabs were collected carefully through a vaginal dilator device to avoid contamination from the vagina. Vaginal and cervical canal swabs were immediately placed in a clean 2 ml DNA LoBind tube containing 0.5 ml sterile saline. Samples were immediately stored on ice and transferred to −80°C freezer within 2 h followed by DNA extraction. All the materials used here were strictly sterilized.

### Laboratory Methods

This study amplified variable regions 3–4 (V3–4) for both vaginal and cervical canal samples. Vaginal and cervical canal swabs were placed in bacteria-free tubes containing 0.5 ml sterile saline. Samples were immediately placed on ice, transferred to −80°C within 2 h, and then stored at −80°C until DNA extraction, followed by PCR amplification. Sequencing was conducted on the MiSeq platform (Illumina) by a validated protocol.

### Statistical Analysis

Bacterial taxa were classified to the genus level, and relative abundance was quantified based on each taxa contribution to an individual sample. Alpha diversity was calculated by Shannon index, and *p* value was calculated by paired *t* tests. OTUs that discriminated between PCOS patients and healthy controls in both sites were analyzed by LEfSe. A LEfSe score of more than 2.0 was considered significant. Canonical correlation analysis (CCA) was used to assess the relevance between clinical characteristics and OTUs (Liu et al., 2019; Sankaran and Holmes, 2019). A phylogenetic study of communities by reconstructing unobserved states (PICRUSt) was used to infer KOs from previously described OTU data. The relative abundance of Kyoto Encyclopedia of Genes and Genomes (KEGG) pathways and modules is summarized from the relative abundance of KOs belonging to these paths and modules.

## Results

### Participants and Amplicon Sequencing Data

Vaginal and cervical canal samples were obtained from 97 women (47 PCOS patients, 50 controls) between December 2018 to June 2019. A total of 97 paired vaginal and cervical canal samples were available for sequencing and analysis. All women recruited here were Chinese, and majority of them were non-smoking and reproductive aged.

Vaginal bacteria 16S rRNA V3–4 gene sequencing obtained a median sequencing read depth of 66,809 reads for each sample (ranging from 51,366 to 82,251). Cervical canal bacteria 16S rRNA gene sequencing obtained a median sequencing read depth of 68,948 reads for each sample (ranging from 56,744 to 81153).

### LGT Microbiomes Composition of PCOS Patients and Healthy Controls

**FIGURE 1:**
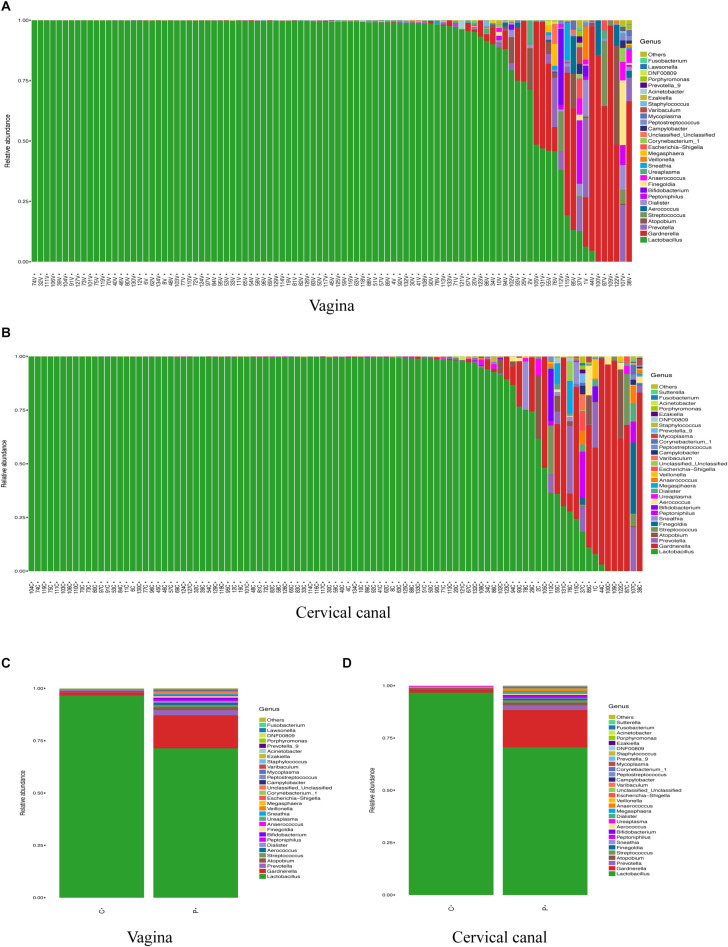
Composition of LGT microbiomes from healthy controls and PCOS patients. General taxa (genus level) composition of vagina **(A)** and cervical canal **(B)** of PCOS patients (*n* = 47) and healthy controls (*n* = 50) are presented, and the main 30 genera are listed. Grouped OTU abundance of vagina **(C)** and cervical canal **(D)** microbiome from PCOS and healthy women are also listed.

Figure 1 shows the most abundant 30 genus in samples for each study object (vagina in Figure 1A ranked by the abundance of *Lactobacillus* and cervical canal in Figure 1B). The most abundant genus in both sites of enrolled patients was *Lactobacillus*, as previously reported. Using canonical correlation analysis, the centroid ellipses of vaginal and cervical canal OTUs were almost overlapped, with a *p* value = 1 (Supplementary Figure S1A), indicating that there were no differences in OTUs between vaginal and cervical canal samples.

Grouped analysis of vaginal and cervical canal microbiome shows that most women from the control group harbored high levels of *Lactobacillus* in both sites (≥90%); however, PCOS patients show a great heterogeneity, with about 30% of patients (*Lactobacillus*-deficient, PCOS-LD subgroup) harboring a society which contains lower levels of *Lactobacillus* (<50%) in the vagina, with some individuals even lacking the *Lactobacillus* genus (Supplementary Figures S1B,C).

### Comparison of Biodiversity of LGT Microbiomes in PCOS and Healthy Controls

**FIGURE 2:**
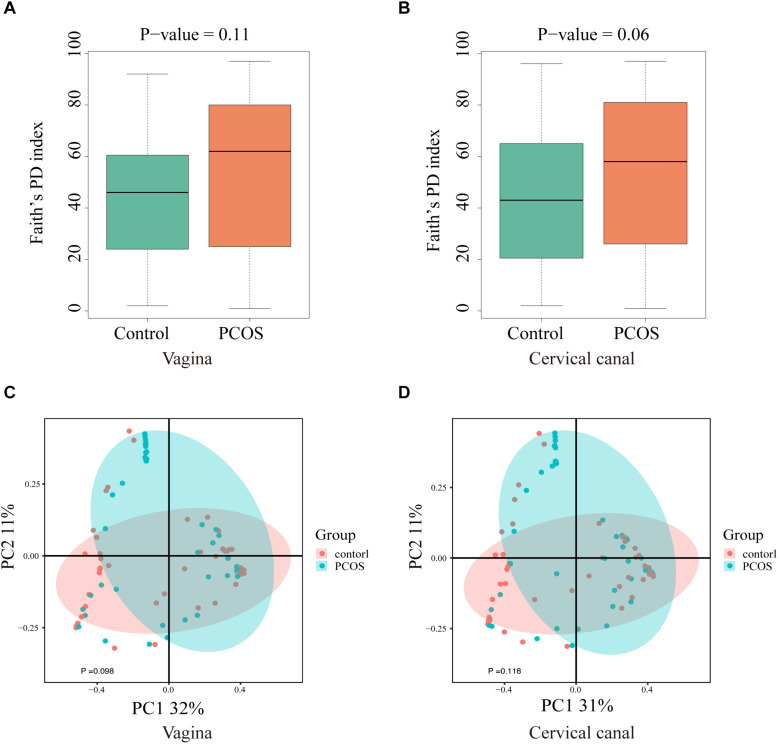
Biodiversity of the LGT microbiomes is increased in PCOS patients. Box plots of the biodiversity in vagina and cervical canal samples from healthy women (*n* = 50), and women diagnosed with PCOS using the Rotterdam criteria (*n* = 47) were presented. α-Diversity was presented by Faith’s PD index: vagina **(A)** and cervical canal **(B)**; β-diversity of microbiome was performed using PCoA analysis: vagina **(C)** and cervical canal **(D)**.

Next, we compared the diversity of microbial composition in LGT between the two groups (Figure 2). The composition of OTUs between PCOS patients and healthy controls suggests that the microbiomes of PCOS patients are more diverse. The bacterial genus content is higher than the healthy controls. Although α-diversity in both sites of the PCOS group appeared to be higher than the control group, the difference between the two groups on both sites was not statistically significant: vagina (*p* = 0.11) and cervical canal (*p* = 0.06). The beta diversity of the PCOS group also seemed to be higher than that in the control group; however, the *p* value still lacked statistical difference: vagina (*p* = 0.098) and cervical canal (*p* = 0.118).

### LGT Taxa Differences Between PCOS and Healthy Controls

**FIGURE 3:**
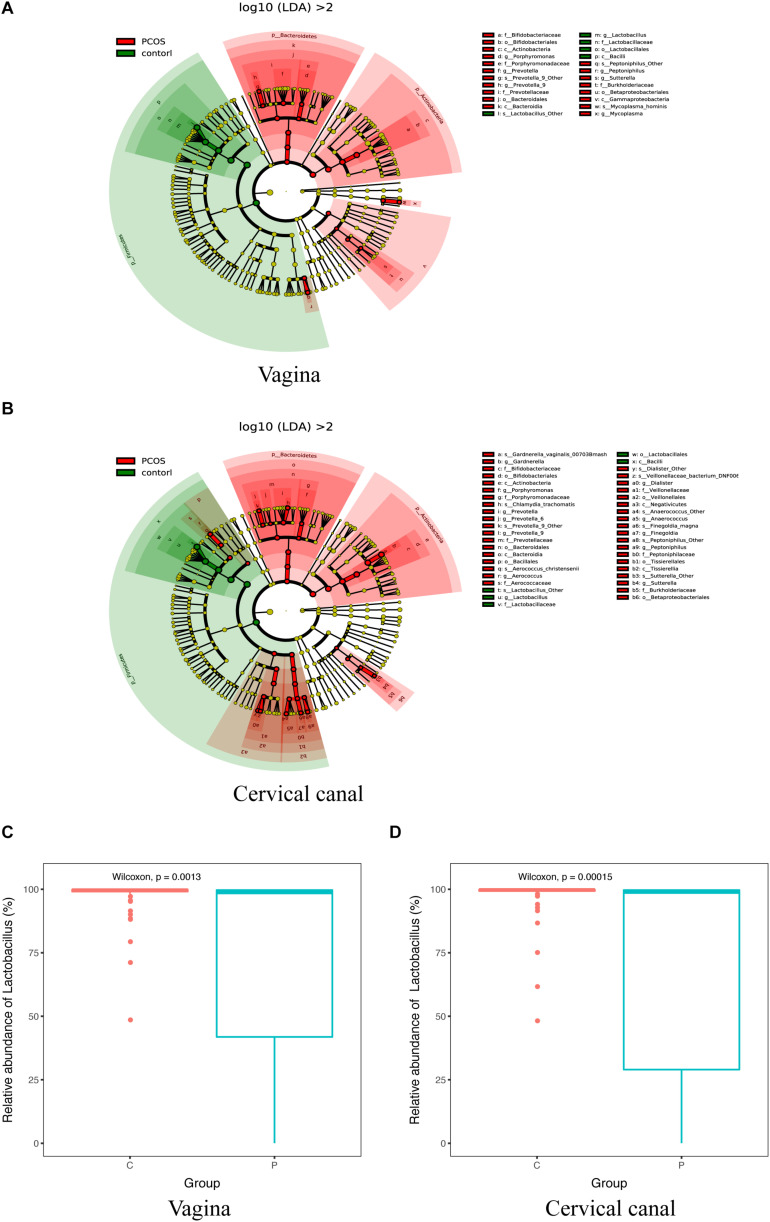
Taxa differed between PCOS and healthy women. 16S-Derived microbial taxa abundance were compared between healthy controls and PCOS patients in both vagina **(A)** and cervical canal **(B)** as analyzed by LEfSe projected as a cladogram and by LDA score (>2). Relative abundance of *Lactobacillus* in both vagina **(C)** and cervical canal **(D)** of PCOS women was significantly reduced compared with the healthy controls, as measured by Wilcoxon test.

By using LEfSe analysis, we found that the relative abundance of several taxa was compared between the PCOS and control groups (Figures 3A,B). *Lactobacillus* (up to the Bacilli class) was significantly reduced in the LGT of the PCOS patients (Figures 3C,D) with a *p* value of 0.0013 (V) and 0.00015 (C), while several other species were more abundant in the PCOS group. *Gardnerella_vaginalis_00703Bmash*, *Chlamydia_trachomatis*, *Prevotella_9_other*, *Aerococcus_christensenii*, and *Dialister_other* were more abundant in the cervical canal of the PCOS patients; whereas, species like *prevotella_9_Other*, *Peptoniphilus_Other*, and *Mycoplasma_hominis* were enriched in the vagina of PCOS patients (LDA score > 2) (Supplementary Figure S2).

### KEGG Functional Analysis

**FIGURE 4:**
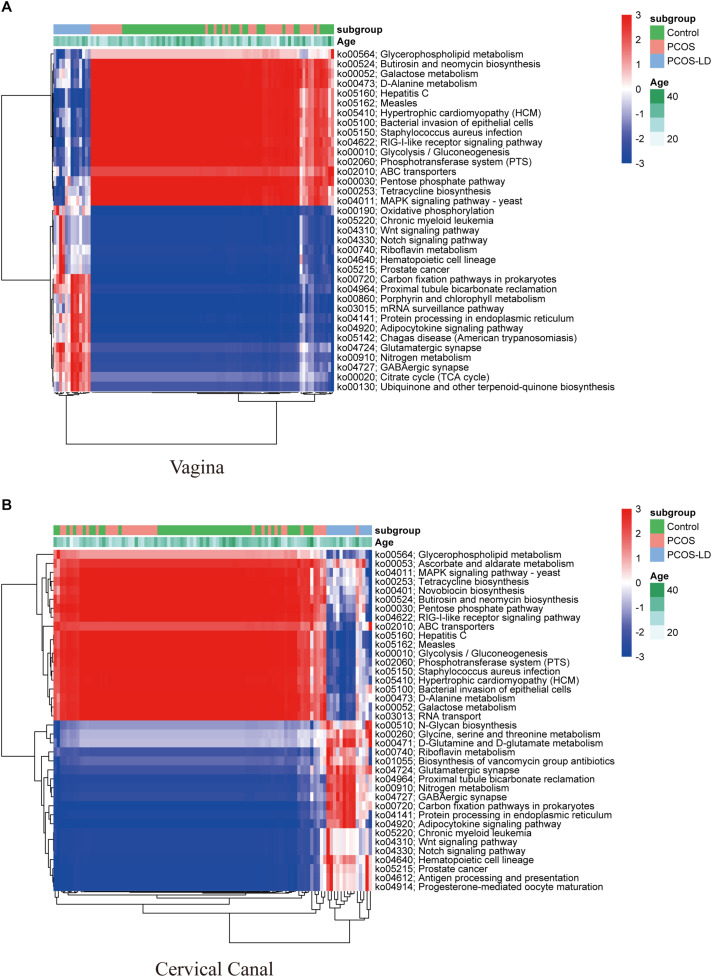
Functional analysis of microbial composition in a total of 97 women’s LGT. KEGG pathways were predicted by the relative abundance of microorganisms in 97 women’s LGT. The dysregulated KEGG pathways in the vagina and cervical canal were selected by Wilcoxon rank-sum test and visualized as a heatmap in panels **(A,B)**, respectively. The pathways and samples were clustered by unsupervised hierarchical clustering.

To further explore the influence of those microbiotas on women’s LGT microenvironment, we also conducted KEGG pathway analysis (Figure 4). Consistent with the microbial composition, seen as higher abundance of pathways for amino acid metabolism, oxidative phosphorylation, and various types of N-glycan biosynthesis in PCOS-LD patients’ genital tract, as those pathways were reported as not conductive to the growth of *Lactobacillus* but is very beneficial to the growth of several potential pathogenic species, such as *Gardnerella* (Chen et al., 2017). Functionally, pathways including glycolysis, glycerophospholipid metabolism, and pentose phosphate were significantly downregulated in the PCOS-LD patients’ genital tract, as these pathways were reported to be associated with proliferative phase or secretory phase in the vagina and endometrium, suggesting that their dysregulation in PCOS patients might be associated with delayed transformation to proliferative and secretory phase and long-term anovulation. Besides, pathways such as antigen processing and presentation, antibiotic biosynthesis, and several other signaling pathways were also observed to be overactivated in PCOS patients, which indicate a probable inflammatory situation in those patients’ genital tract.

### Correlation Between Microbiome and Clinical Index

To examine the clinical relevance of the microorganisms, we also collected clinical information on the studied cases (Table 1) and analyzed their correlation with microbial composition through CCA, and the results are shown in Supplementary Figure S3. Among the data of menstrual cycle, BMI, Testo level, past vaginal inflammation history, and abortion history, the flora that enriched in PCOS patients’ LGT were most correlated to menstrual cycle and vaginosis history.

**TABLE 1:** Clinical characteristics of study participants.

	Control (*n* = 50)	PCOS (*n* = 47)	*p* value 1
Body mass index	21.8 ± 2.7	24.4 ± 3.8	0.0006
Menses per year	12.5 ± 1.1	6.4 ± 1.3	<0.0001
Testosterone (nmol/L)	1.7 ± 0.7	2.3 ± 0.7	0.0001
**Smoking**			
Yes	2 (4.0%)	2 (4.3%)	ns
No	48 (96.0%)	45 (95.7%)	ns
**Alcohol intaking**			
Yes	1 (2.0%)	2 (4.3%)	ns
No	49 (98.0%)	45 (95.7%)	ns
Sexual frequency/month	5.0 ± 2.7	4.5 ± 3.0	ns
Hyperandrogenism	0	14	<0.0001
Oligomenorrhea	0	47	<0.0001
Polycystic ovarian morphology	0	47	<0.0001

*Data is presented as mean ± SD. Non-normal data was ranked and analyzed by Mann-Whitney U test.*

## Discussion

This is the first study to explore the lower genital tract microbiome composition of PCOS patients. A great heterogeneity of microbiome composition was observed both in the vagina and cervical canal of PCOS patients, whereas PCOS patients’ LGT carried a significantly reduced proportion of *Lactobacillus* but an increased proportion of several potential pathogenic taxa, such as *Gardnerella_vaginalis_00703Bmash*, *Chlamydia_trachomatis*, and *Prevotella_9_other*. The vaginal and cervical canal microbiome from the same person showed great similarity. Taxa enriched in PCOS patients’ genital tract also showed a strong correlation with menstrual cycle.

The vaginal microbiome can be principally affected by the effects of estrogen and progesterone on vaginal epithelial cells, the predominance of *Lactobacillus*, low PH, and several other factors, such as sexual activity, menses, and use of antimicrobial agents (Aroutcheva et al., 2001; Chen et al., 2017; Buchta, 2018). The microorganisms that live in the upper genital tract are mostly considered to be originate from the ascending of vaginal microbiome, which can also affect the sex hormones and menses (Rampersaud et al., 2012; Khan et al., 2016; Moreno and Franasiak, 2017). As PCOS patients are characterized by an irregular mense cycle and sex hormone disorder (Trikudanathan, 2015; Thackray, 2019), in this study, we found that the lower reproductive tract microorganisms of those patients are significantly altered. As PCOS patients are often plagued by infertility, abortion, fetal arrest, preterm birth, and several other adverse reproductive outcomes, we doubt if there is a certain correlation between the compositions of microorganisms in PCOS women’s LGT and these adverse reproductive phenotypes.

Consistent with the low diversity of genital tract microbiome in healthy reproductive-aged women, *Lactobacillus* spp. is the most abundant bacteria in women’s LGT, as reported (Ravel et al., 2010; Stumpf et al., 2013). However, we found a significantly reduced level of *Lactobacillus* spp. in PCOS patients’ lower genital tract, especially in the PCOS-LD group, whereas several other potential pathogens including *Gardnerella*, *Prevotella*, *Veillonellaceae*, *Streptococcus*, and *Dialister* species were enriched. A reduced level of *Lactobacillus* spp. in women’s reproductive tract are tightly associated with a high risk of vaginosis, infertility, abortion, stillbirth, preterm labor, recurrent implantation failure, and many other adverse pregnancy outcomes (Macklaim et al., 2013; Paavonen and Brunham, 2018; Al-Memar et al., 2019; Gupta et al., 2019; Koedooder et al., 2019; Peelen et al., 2019; Pekmezovic et al., 2019). *Gardnerella* and *Prevotella* species have been reported to be closely related to bacterial vaginosis (BV), which is a big threat to women’s reproductive health and easy relapse (Pepin et al., 2011; Coudray and Madhivanan, 2020). As in about half of women with BV, *Gardnerella vaginalis* can also be detected in their endometrium, which may have adverse effects on the procedure of embryo implantation and even the growth of fetus (Swidsinski et al., 2013; Vestby et al., 2020). In our study, *Gardnerella vaginalis* species was significantly enriched in both the vagina and cervix of PCOS patients. We doubt whether this flora may also be enriched in those patients’ uterus. Besides, C*hlamydia trachomatis*, *Dialister*, *Veillonella*, *Streptococcus*, and *Peptoniphilus* species are also correlated with high Nugent scores, and higher proportion of these bacteria are prone to sexually transmitted diseases (STD) and BV (Shannon et al., 2017; Di Pietro et al., 2018; Dabee et al., 2019). Moreover, the levels of *Prevotella* and *Dialister* species in the vagina were higher in patients with preterm birth (Fettweis et al., 2019; Lokken et al., 2020). However, we excluded vaginitis from each participant we enrolled in this study. The taxa differences in these patients may represent a less healthy environment of LGT. Collectively, the increased diversity among genital tract microbiome lowers the percentage of *Lactobacillus* spp., and together with the implantation of those potentially pathogenic bacteria in women’s lower genital tract, several disorders in women’s reproductive system have been shown to be strongly associated with the following: infertility, abortion, preterm birth, and low IVF transplantation rate (Mitchell et al., 2015; Anahtar et al., 2018; Benner et al., 2018; Suff et al., 2018; Peelen et al., 2019), which are also common symptoms of PCOS patients. All these evidences indicated that there is a significant correlation between the microbial characteristics of the LGT and the clinical reproductive symptoms of PCOS patients, which is a completely new concept for clinicians and can also provide new ideas and directions for improving the pregnancy outcome of PCOS patients.

In recent years, scientists have paid more attention to the microenvironment of women’s reproductive tract, as increasing evidences have revealed a close association of microenvironment with reproductive health and the long-term health of offspring (Rampersaud et al., 2012; Franasiak and Scott, 2015; Yarbrough et al., 2015). Unlike gut microbiome, a healthy genital tract microbiome is characterized by *Lactobacillus* spp. dominance and a relatively low biodiversity (Ravel et al., 2010). Most *Lactobacillus* spp. can utilize free glycogen and then convert them to lactic acid, which can keep the vagina acidic (Ghartey et al., 2014; Mirmonsef et al., 2014). Higher microbiota diversity was associated with an elevated PH and diverse types of metabolites, such as short-chain fatty acids (SCFAs), succinate instead of lactic acid, acetate, and butyrate. Those metabolites may also play a key role in the pathogenic procedure of reproductive issues (Newman and Koren, 2017; Anahtar et al., 2018). In this study, the KEGG analysis also showed that pathways, such as N-glycan biosynthesis and oxidative phosphorylation, were predicted to be overactivated in the LGT society of the PCOS-LD group, as is associated with the *Lactobacillus*-deficient society. The vagino-uterine microbiota probably varies during the menstrual cycle (Gajer et al., 2012), while the proliferative period seems to be related to the increase of bacterial proliferation in the vagina and endometrium (Chen et al., 2017), however, the pathways correlated to the proliferative or secretory phase were both downregulated in the PCOS-LD group, as PCOS patients usually present an irregular menstrual cycle, which may be the reason why the microorganism in the reproductive tract of PCOS patients is more easier to alter. As potential pathogenic taxa such as *Gardnerella* and *Prevotella* were enriched in PCOS patients, it might cause disrupted immune homeostasis in the genital tract. Consistently, some inflammation-related pathways such as antigen processing and presentation, antibiotic biosynthesis, and several signaling pathways were also overactivated in the PCOS-LD patients, suggesting that the immune response were overactivated in those patients’ genital tract, which is harmful to women’s reproductive health, and thereby affect the situation of the uterus (Anahtar et al., 2018).

Lower genital tract microbiome could be affected by diverse factors, such as menopause, hormones, age, hygienic habits, etc (Muhleisen and Herbst-Kralovetz, 2016). Prepuberty females and postmenopausal women’s LGT were colonized with diverse kinds of microbes, which indicates that menopause and sex hormones (especially estrogen) may be extremely important for the composition of LGT microbes. Additionally, pregnant women’s vagina was shown to carry higher levels of *Lactobacillus*, which may be affected by the increased level of estrogen and progesterone, weight gain, and immune system modulations (Newman and Koren, 2017). While most PCOS patients are often accompanied by irregular menstruation and abnormal hormone levels, we believe that this may be the main factors leading to the change of LGT in those patients, especially the significant reduction of *Lactobacillus*. Consistently, by analyzing the correlation of LGT microbiome with clinical indexes, we found that the menstrual cycle length was most strongly related to the composition of PCOS patients’ LGT microbiomes. Since the normal menstrual cycle under the action of estrogen and progesterone causes periodic changes in the epidermal cells of the reproductive tract, this process may play a critical role in maintaining the microenvironment of the genital tract. However, the mechanisms underlying this phenomenon are unclear.

## Conclusion

In the present study, we systematically analyzed the lower genital microbiome profiles in PCOS women and healthy controls. The overall microbiome of PCOS patients was compared from that of healthy controls. Particularly, PCOS patients exhibited significantly reduced *Lactobacillus* spp. in both cervical and vaginal canals. The taxa enriched in the PCOS group were strongly related to the adverse reproductive outcome of PCOS patients, as reported. The KEGG pathway analysis provided a potential link between the dysregulated bacteria and the pathways that they were potentially involved in. In addition, the length of menstrual cycle seems correlated to most taxa enriched in the PCOS group.

In summary, this study does not only improve our understanding of overall composition of the LGT microorganisms that lived in PCOS patients but also provided evidences of PCOS-related pathogens and underlying mechanisms for further research.

## Data Availability Statement

The datasets presented in this study can be found in online repositories. The names of the repository/repositories and accession number(s) can be found below: https://biosino.org/node/search, OEP000469.

## Ethics Statement

The studies involving human participants were reviewed and approved by International Peace Maternity and Child Health Hospital. The patients/participants provided their written informed consent to participate in this study.

## Author Contributions

LJ, JS, YT, and HH designed the trial. GD, YaW, YG, and HG recruited the patients and collected the specimens, XL, YiW, JX, and GZ interpreted the data and critically reviewed the article. All authors contributed to the article and approved the submitted version.

## Conflict of Interest

The authors declare that the research was conducted in the absence of any commercial or financial relationships that could be construed as a potential conflict of interest.

## Supplementary Material

FIGURE S1 Characterization of LGT microbiome in a total of 97 women’s LGT. PCoA analysis of microbiomes between the two sites (vagina and cervical canal) is shown in **(A)**. Proportion of *Lactobacillus* in vagina microbiome is showed in **(B)**. Differences of α-diversity of vagina and cervical canal microbiome between healthy controls (*n* = 50), PCOS (non-LD) (*n* = 34), and PCOS-LD (*n* = 13) measured by Faith’s PD score as shown in **(C)**.

FIGURE S2 Taxa differences of LGT microbiome between PCOS and healthy women. 16S-Derived microbial taxa were identified as differentially abundant between healthy controls and PCOS patients in both vagina **(A)** and cervical canal **(B)** and were analyzed by LEfSe projected with an LDA score.

FIGURE S3 Canonical correlation between OTUs and clinical indexes. The correlation between the first two typical axes of OTU linear combination and the main relevant clinical indexes was plotted.
