# Corrigendum: Comparative Analysis of Lower Genital Tract Microbiome Between PCOS and Healthy Women

**DOI:** 10.3389/fphys.2020.635088

**Published:** 2021-01-18

**Authors:** Yaoyao Tu, Guangyong Zheng, Guolian Ding, Yanting Wu, Ji Xi, Yingzhou Ge, Hangchao Gu, Yingyu Wang, Jianzhong Sheng, Xinmei Liu, Li Jin, Hefeng Huang

**Affiliations:** ^1^Department of Obstetrics and Gynecology, International Peace Maternity and Child Health Hospital, Shanghai Jiao Tong University, Shanghai, China; ^2^Shanghai Key Laboratory of Embryo Original Diseases, Shanghai, China; ^3^Bio-Med Big Data Center, CAS Key Laboratory of Computational Biology, CAS-MPG Partner Institute for Computational Biology, Shanghai Institute of Nutrition and Health, Shanghai Institutes for Biological Sciences, Chinese Academy of Sciences, Shanghai, China; ^4^Key Laboratory of Reproductive Genetics, Ministry of Education, Zhejiang University, Hangzhou, China; ^5^Department of Pathology and Pathophysiology, School of Medicine, Zhejiang University, Hangzhou, China

**Keywords:** *Gardnerella*, *Lactobacillus*, lower genital tract (LGT), microbiome, PCOS

**Li Jin** and **Xinmei Liu** was not included as an co-corresponding author in the published article. The corrected New Author Contributions Statement appears below.

**New Author Contributions Statement**

†These authors have contributed equally to this work and share first authorship

^*^Xinmei Liu, Li Jin, and Hefeng Huang are co-corresponding authors.

The authors apologize for this error and state that this does not change the scientific conclusions of the article in any way. The original article has been updated.

