# Supplementary figures and images for: Comparative Analysis of Lower Genital Tract Microbiome Between PCOS and Healthy Women
Source: Front Physiol. 2020 Sep 8;11:1108. doi: 10.3389/fphys.2020.01108 (PMC7506141; doi:10.3389/fphys.2020.01108)

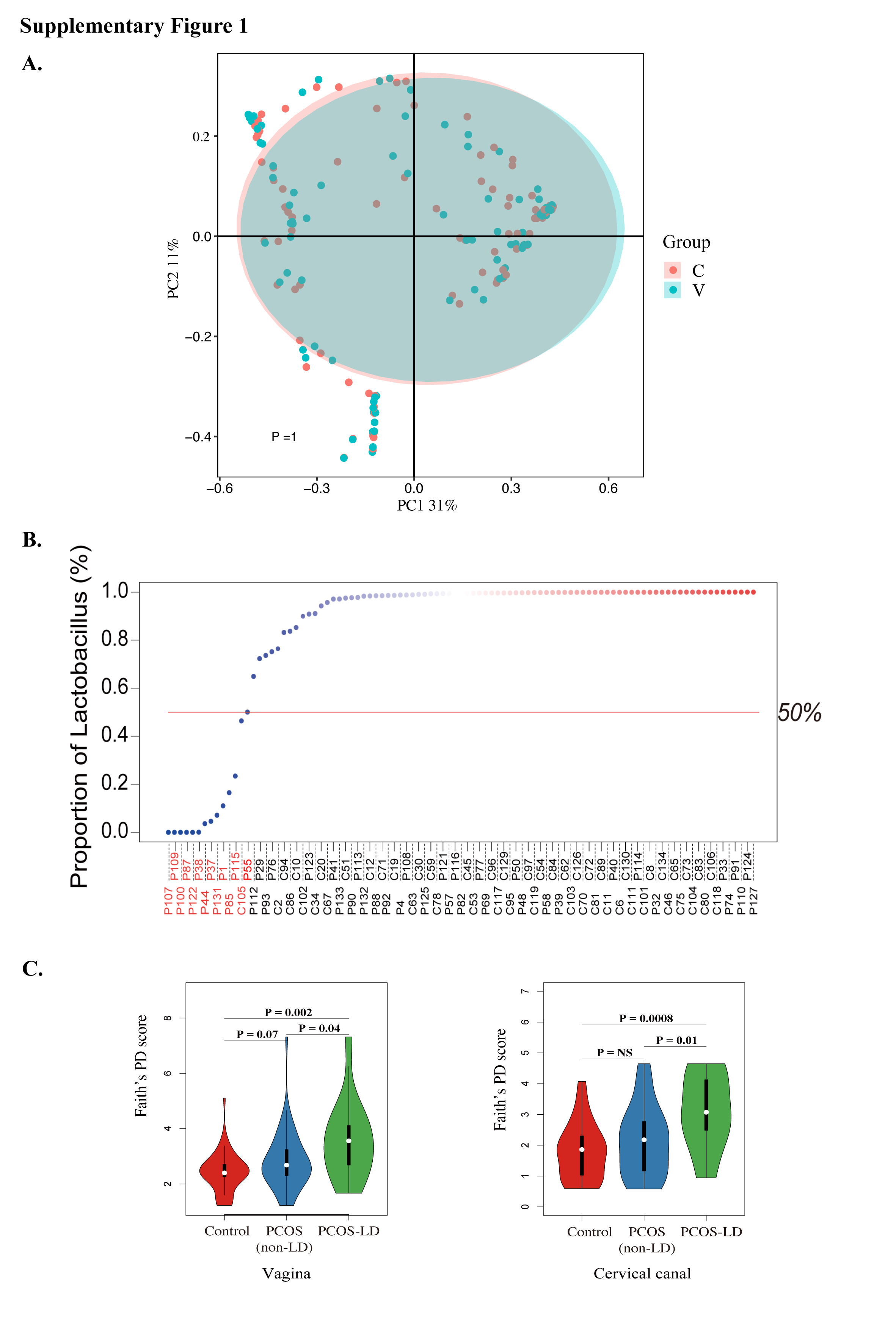

Supplement: FIGURE S1 — Characterization of LGT microbiome in a total of 97 women’s LGT. PCoA analysis of microbiomes between the two sites (vagina and cervical canal) is shown in (A). Proportion of Lactobacillus in vagina microbiome is showed in (B). Differences of α-diversity of vagina and cervical canal microbiome between healthy controls (n = 50), PCOS (non-LD) (n = 34), and PCOS-LD (n = 13) measured by Faith’s PD score as shown in (C). [file Image_1.TIF]

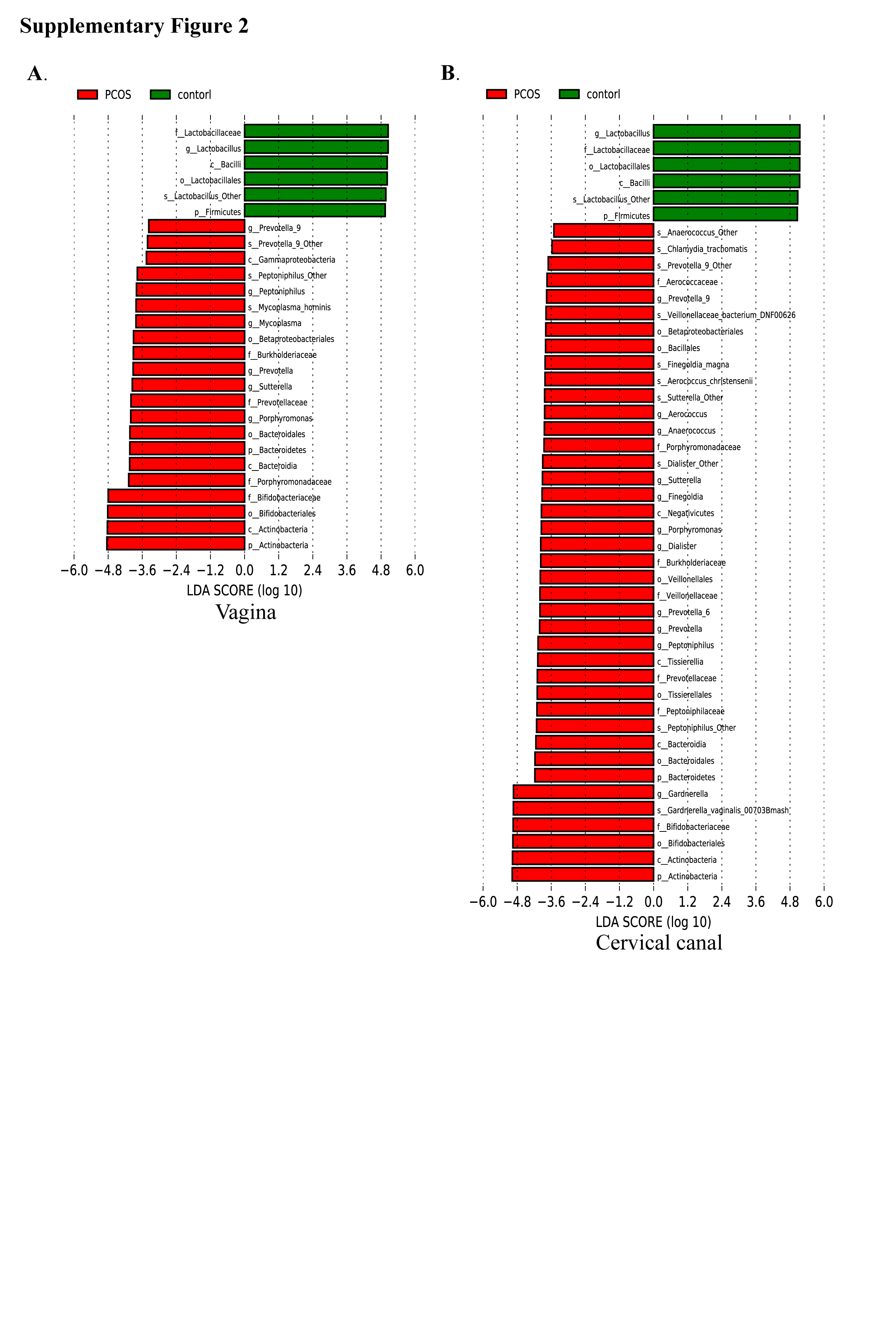

Supplement: FIGURE S2 — Taxa differences of LGT microbiome between PCOS and healthy women. 16S-Derived microbial taxa were identified as differentially abundant between healthy controls and PCOS patients in both vagina (A) and cervical canal (B) and were analyzed by LEfSe projected with an LDA score. [file Image_2.TIF]

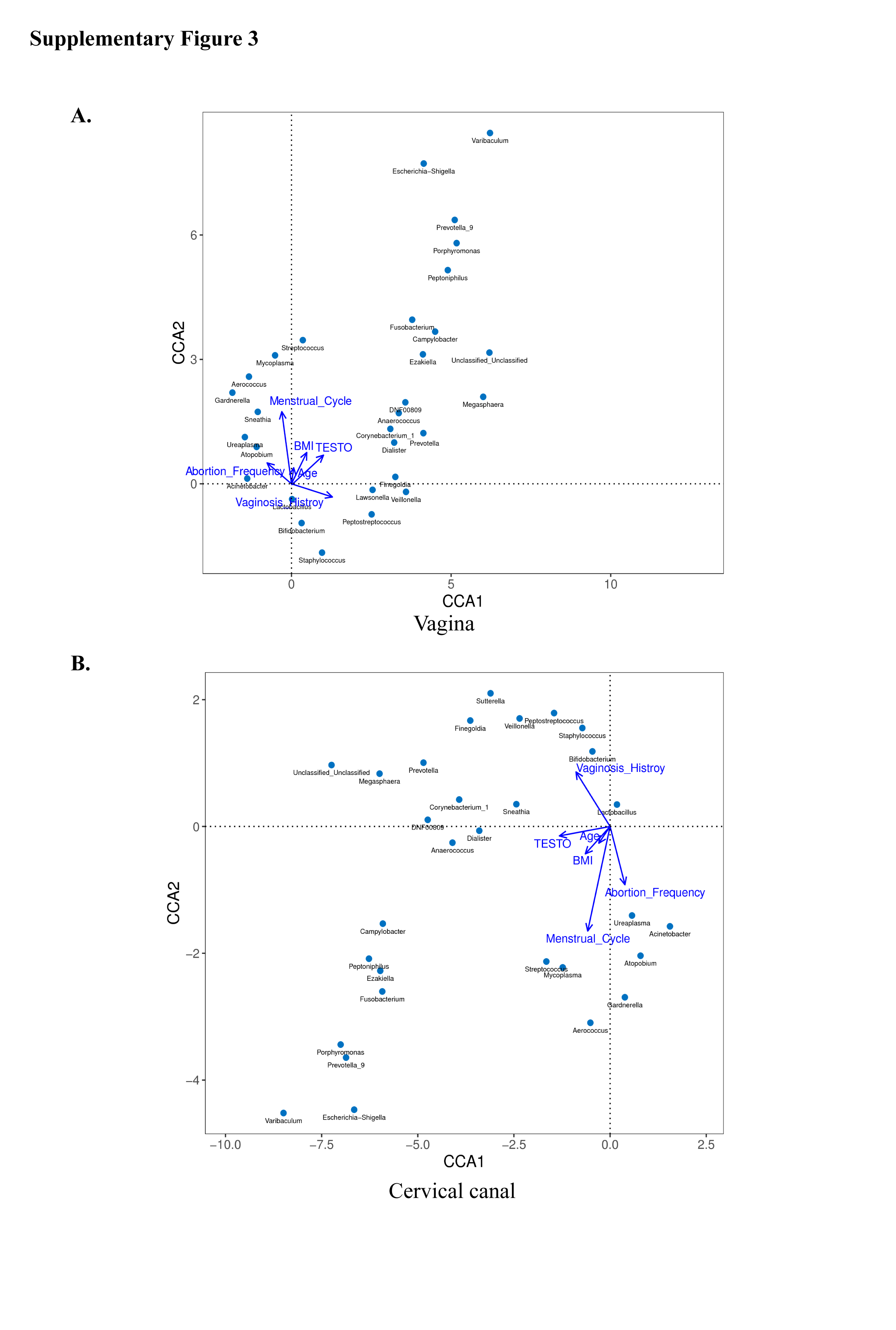

Supplement: FIGURE S3 — Canonical correlation between OTUs and clinical indexes. The correlation between the first two typical axes of OTU linear combination and the main relevant clinical indexes was plotted. [file Image_3.TIF]
